# Reactivation of microRNA-506 inhibits gastric carcinoma cell metastasis through ZEB2

**DOI:** 10.18632/aging.101877

**Published:** 2019-03-28

**Authors:** Gui-Jun Wang, Bao-Ping Jiao, Yang-Jun Liu, Yan-Rong Li, Bei-Bei Deng

**Affiliations:** 1 Department of General Surgery, The First Affiliated Hospital of Jinzhou Medical University, Jinzhou 121000, China; 2 Department of Gastroenterology, The First Affiliated Hospital of Jinzhou Medical University, Jinzhou 121000, China; 3 Department of Clinical Laboratory, The First Affiliated Hospital of Jinzhou Medical University, Jinzhou 121000, China

**Keywords:** microRNA, miRNA microarray, miR-506, gastric carcinoma, ZEB2

## Abstract

MicroRNAs (miRNAs) are frequently dysregulated in a variety of human cancers, including gastric carcinoma. To improve our understanding of the role of miRNAs in gastric carcinoma and potential identify novel biomarkers or therapeutic agents, we performed microarray analysis to identify differentially expressed miRNAs in gastric carcinoma, compared with paired non-cancerous gastric tissues. We identified significantly differentially expressed miRNAs in gastric carcinoma tissues, including miR-506. We validated the microarray results by quantitative reverse transcription polymerase chain reaction in 26 specimens and confirmed significant downregulation of miR-506 in gastric carcinoma. Bioinformatics analysis predicted ZEB2 (zinc finger E-box-binding homeobox 2) as a potential target of miR-506. MiR-506 levels and ZEB2 levels were inversely correlated in gastric carcinoma, and low miR-506 levels in gastric carcinoma were associated with poor prognosis. Overexpression of miR-506 in gastric carcinoma cells significantly inhibited cell migration and invasion, while depletion of miR-506 in gastric carcinoma cells significantly increased cell migration and invasion. Transplantation of miR-506-overexpressing gastric carcinoma cells developed significantly smaller tumor, compared to the control. Thus, our results suggest that miR-506 may function as a tumor suppressor and targets and inhibits ZEB2 in gastric carcinoma.

## INTRODUCTION

Gastric carcinoma contributes to a majority of cancer-related death in China [1]. Although surgical removal of the tumor-baring stomach remains the most effective treatment for gastric carcinoma, more than half of Gastric carcinoma patients are diagnosed at later stages, when surgical therapy is not sufficient for curing the disease due to extensive growth and metastasis of the primary tumor [2]. Therefore, better understanding of the molecular mechanisms underlying the growth and metastasis of gastric carcinoma is critical for the identification of biomarkers and novel therapeutic agents for gastric carcinoma. Recently, it is noticed that deregulation of microRNAs (miRNAs) frequently occur in a variety of human cancers, including gastric carcinoma [3]. However, our understanding on this issue remains limited.

MicroRNAs (miRNAs) are small non-coding RNA molecules that regulate gene expression at the post-transcriptional level through interaction with the 3′-untranslated regions (UTRs) of target mRNAs [4]. MiRNAs are expressed in a tissue-specific manner and play important roles in various cellular functions, including cell proliferation and apoptosis. Increasing studies have also established a critical role for miRNAs in cancer development [5]. Aberrant expression of miRNAs has been demonstrated in tumor tissues compared with matched normal tissues. miRNAs can function as tumor suppressors or oncogenes during the process of tumorigenesis, indicating their potential as biomarkers for diagnosis and therapeutic targets [6]. Recent reports have identified a few miRNAs that may contribute to the development and progression of gastric carcinoma by promoting oncogene expression or inhibiting tumor suppressor genes [7]. However, the identification of other differentially regulated miRNAs in gastric carcinoma is required to help further our understanding of the mechanisms underlying gastric carcinoma development and may potentially lead to more therapeutic targets or biomarkers for gastric carcinoma.

In this study, we performed microarray analysis to identify differentially expressed miRNAs in gastric carcinoma tissue and evaluated the potential roles and underlying mechanisms of one of the identified miRNAs, miR-506, in gastric carcinoma invasion and metastasis. We validated the microarray results by quantitative reverse transcription polymerase chain reaction in 26 specimens and confirmed significant downregulation of miR-506 in gastric carcinoma. Bioinformatics analysis predicted ZEB2 (zinc finger E-box-binding homeobox 2) as a potential target of miR-506. MiR-506 levels and ZEB2 levels were inversely correlated in gastric carcinoma, and low miR-506 levels in gastric carcinoma were associated with poor prognosis. Overexpression of miR-506 in gastric carcinoma cells significantly inhibited cell migration and invasion, while depletion of miR-506 in gastric carcinoma cells significantly increased cell migration and invasion. Transplantation of miR-506-overexpressing gastric carcinoma cells developed significantly smaller tumor, compared to the control.

## RESULTS

### MiR-506 and ZEB2 expression in gastric carcinoma

**Figure 1:**
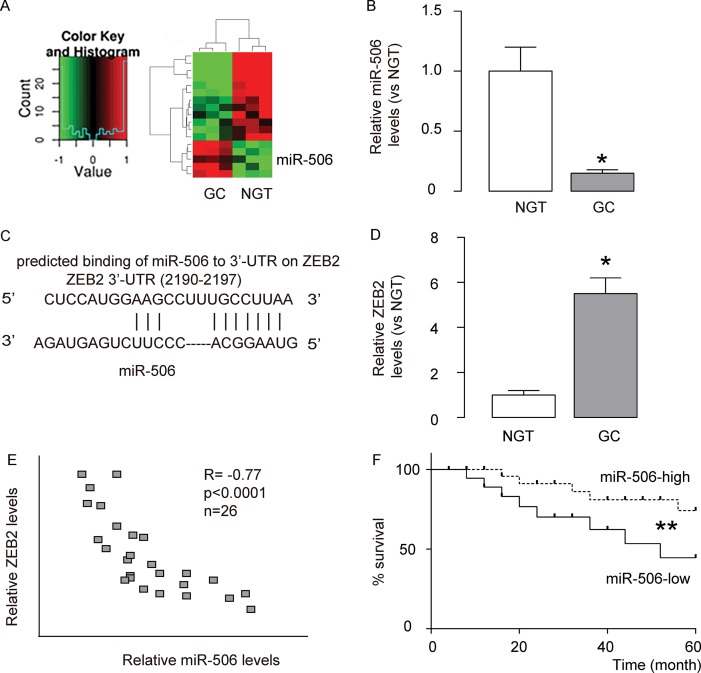
**MiR-506 and ZEB2 expression in gastric carcinoma.** (**A**) We performed miRNA microarray analysis to compare the miRNA expression profiles between gastric carcinoma tissues (GC) and paired non-cancerous gastric tissue (NGT). We set the cut-off level as fold change > 2 and P value < 0.05. The original intensities data were normalized using the Quantile method. The microarray analysis identified some unique differentially expressed miRNAs. (**B**) RT-qPCR for miR-506 in GC versus NGT. (**C**) Bioinformatics analysis showed that miR-506 targets ZEB2, which is a well-known factor that regulates cell invasiveness and migration, (**D**) Western blot for ZEB2 in GC versus NGT. (**E**) Correlation between miR-506 and ZEB2 in 26 gastric carcinoma specimens (ɤ= -0.77, p<0.0001). (**F**) The all 26 patients included in this study were followed up for 5 years for the overall survival. The median value of miR-506 in the 26 patients was used as a cutoff point to separate the total samples into miR-506-high group (n=13) and miR-506-low group (n=13). The Kaplan-Meier curves for the overall 5-year survival of these patients were shown. *p<0.05. **p<0.01. N=26.

We performed miRNA microarray analysis to compare the miRNA expression profiles between gastric carcinoma tissues and paired non-cancerous gastric tissue (NGT). We set the cut-off level as fold change > 2 and P value < 0.05. The original intensities data were normalized using the Quantile method. The microarray analysis identified some unique differentially expressed miRNAs (Figure 1A). Among these differentially expressed miRNAs (miR-191, miR-1292, miR-200b, miR-221, miR-18a, Let-71, miR-133a, miR-192, miR-372, miR-421, miR-181c, miR-199, miR-506), we put specific focus on miR-506, after validation of its expression by RT-qPCR (Figure 1B), since by bioinformatics analysis, miR-506 was shown to have a target, ZEB2, which is a well-known factor that regulates cell invasiveness and migration (Figure 1C). Moreover, in the gastric carcinoma samples, we detected significantly higher levels of ZEB2, compared to NGT (Figure 1D). To assess the relationship between miR-506 and ZEB2 in gastric carcinoma, we examined their correlation using the 26 gastric carcinoma specimens. A strong inverse correlation was detected between miR-506 and ZEB2 levels (Figure 1E, ɤ= -0.77, p<0.0001). suggesting presence of a causal link between miR-506 and ZEB2 in gastric carcinoma. The all 26 patients included in this study were followed up for 5 years for the overall survival. The median value of miR-506 in the 26 patients was used as a cutoff point to separate the total samples into miR-506-high group (n=13) and miR-506-low group (n=13). The Kaplan-Meier curves for the overall 5-year survival of these patients showed a significantly poorer survival of miR-506-low patients (Figure 1F). Thus, gastric carcinoma with low miR-506 expression is associated with poor overall survival.

### MiR-506 targets 3’-UTR of ZEB2 mRNA to inhibit its expression

**Figure 2:**
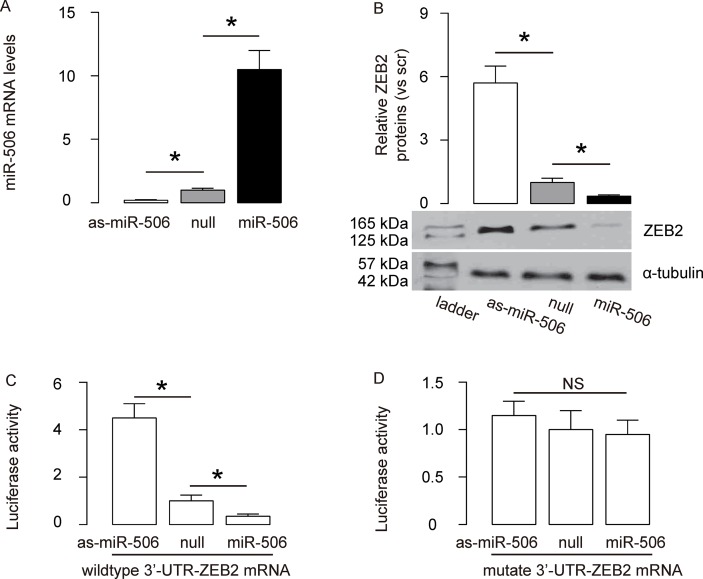
**MiR-506 targets 3’-UTR of ZEB2 mRNA to inhibit its expression.** (**A**) We either overexpressed miR-506, or inhibited miR-506 in AGS cells by transfection of the cells with a miR-506-expressing plasmid (miR-506), or with a plasmid carrying an antisense for miR-506 (as-miR-506). The AGS cells were also transfected with a plasmid carrying a null sequence as a control (null). RT-qPCR for miR-506 was done in the transfected cells. (**B**) Western blot for ZEB2 in transfected AGS cells. (**C–D**) MiR-506-modified AGS cells were then transfected with wildtype (C) or mutate (D) ZEB2-3’-UTR luciferase-reporter plasmid, after which the luciferase activities were quantified. *p<0.05. NS: non-significant. N=5.

Since our data suggest a relationship between miR-506 and ZEB2 in gastric carcinoma, and since bioinformatics analysis suggests ZEB2 as a target for miR-506, we examined this regulatory axis with a human gastric carcinoma cell line, AGS. We either overexpressed miR-506, or inhibited miR-506 in AGS cells by transfection of the cells with a miR-506-expressing plasmid (miR-506), or with a plasmid carrying an antisense for miR-506 (as-miR-506). The AGS cells were also transfected with a plasmid carrying a null sequence as a control (null). The overexpression or depletion of miR-506 in AGS cells by transfection with these plasmids was confirmed by RT-qPCR (Figure 2A). The protein levels of ZEB2 in AGS cells by transfection with these plasmids was examined by Western blot, showing that overexpression of miR-506 in AGS cells significantly decreased ZEB2 protein, while depletion of ZEB2 in AGS cells significantly increased ZEB2 protein (Figure 2B). MiR-506-modified AGS cells were then transfected with wildtype (Figure 2C) or mutate (Figure 2D) ZEB2-3’-UTR luciferase-reporter plasmid, after which the luciferase activities were quantified. The data suggest that miR-506 targets 3’-UTR of ZEB2 mRNA to inhibit its translation (Figure 2C–2D).

### MiR-506 inhibits migration and invasion of gastric carcinoma cells in vitro

**Figure 3:**
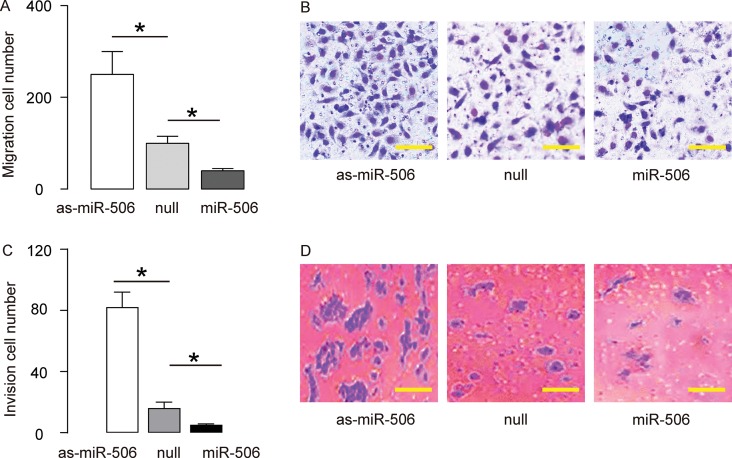
**MiR-506 inhibits migration and invasion of gastric carcinoma cells in vitro**. To assess the function of miR-506 in gastric carcinoma migration and invasion, we challenged the transfected cells in a cell migration assay and a cell invasion assay. (**A–B**) A cell migration assay, shown by quantification (A), and by representative images (B). (**C–D**) A cell invasion assay, shown by quantification (C), and by representative images (D). *p<0.05. N=5. In B, scale bars are 20 µm and in D, scale bars are 50 µm.

To assess the function of miR-506 in gastric carcinoma migration and invasion, we challenged the transfected cells in a cell migration assay and a cell invasion assay. In a cell migration assay, we found that overexpression of miR-506 significantly reduced the migrated cell number, while depletion of miR-506 significantly increases the migrated cell number, shown by quantification (Figure 3A), and by representative images (Figure 3B). In a cell invasion assay, we found that overexpression of miR-506 significantly reduced the invaded cell number, while depletion of miR-506 significantly increases the invaded cell number, shown by quantification (Figure 3C), and by representative images (Figure 3D). Together, these data suggest that miR-506 inhibits migration and invasion of gastric carcinoma cells in vitro.

### Re-activation of miR-506 inhibits the gastric carcinoma xenograft tumor growth in vivo

**Figure 4:**
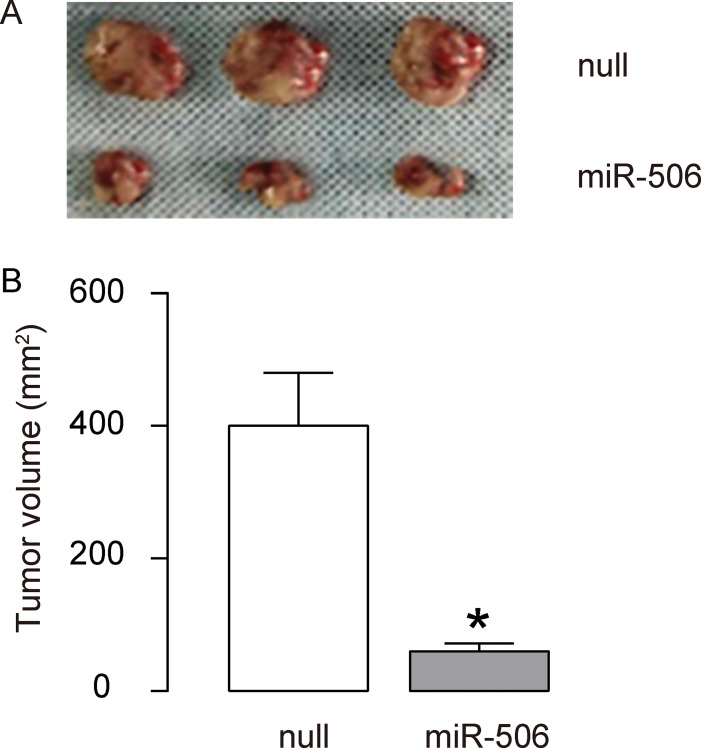
**Re-activation of miR-506 inhibits the gastric carcinoma xenograft tumor growth in vivo.** (**A**–**B**) To further assess the function of miR-506 in gastric carcinoma growth in vivo, we used a xenograft model in which null or miR-506-transfected AGS cells were transplanted subcutaneously into nude mice. The tumor size was determined at 30 days after transplantation. The results were shown by representative images (A) and by quantification (B). *p<0.05. N=3.

**Figure 5:**
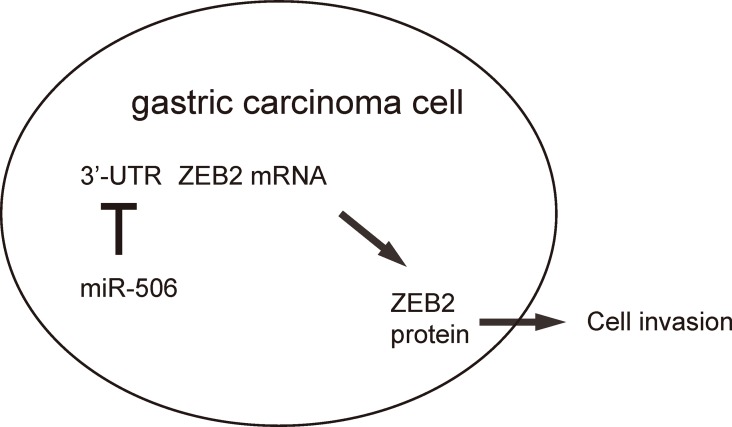
**Schematic of the study.** miR-506 may function as a tumor suppressor by targeting and suppressing ZEB2 protein translation in gastric carcinoma.

To further assess the function of miR-506 in gastric carcinoma growth in vivo, we used a xenograft model in which null or miR-506-transfected AGS cells were transplanted subcutaneously into nude mice. The tumor size was determined at 30 days after transplantation. The results showed that miR-506-overexpression significantly reduced the size of tumor, compare to the control group, shown by representative images (Figure 4A) and by quantification (Figure 4B). Hence, re-activation of miR-506 inhibits the gastric carcinoma xenograft tumor growth in vivo. Our findings here were thus summarized in a schematic, showing that that miR-506 may function as a tumor suppressor by targeting and suppressing ZEB2 protein translation in gastric carcinoma (Figure 5).

## DISCUSSION

MiRNAs represent a class of noncoding RNAs that participate in multiple biological processes including cell differentiation, proliferation and apoptosis [6]. Studies have linked miRNA function with cancer development, and thus clarifying the mechanisms of miRNAs in the progression of cancer has become an area of intense investigation [6]. A growing number of studies has shown that dysregulation of miRNAs is involved in gastric carcinoma progression, demonstrating a potential function for miRNAs in the occurrence and development of gastric carcinoma [7]. For instance, as early as in 2000, a report that investigated microRNA abnormalities in gastric carcinogenesis showed downregulation of miR-200 family members specifically in EBV-associated gastric carcinoma, as compared with that in EBV-negative carcinoma [8]. Interestingly, downregulation of the miR-200 family was found, accompanied by the loss of cell adhesion, reduction of E-cadherin expression, and upregulation of ZEB1 and ZEB2, consistent with the findings in the current study. However, that previous work, although being pioneering, does not demonstrate a direct regulation between miR-2000 members with these genes associated with cell invasion and migration. Here, our study should be a good follow-up of that previous work, by showing the role of the regulatory axis miR-506/ZEB2.

In the current study, we identified several novel miRNAs that have not been detected before in gastric carcinoma and that may play a role during pathogenesis of gastric carcinoma. MiR-506 is frequently downregulated and plays anti-tumor roles in multiple cancer types. For instance, in 2011, Tong et al. showed that miR-506 targets and suppresses PPARalpha, which mediates the resistance to hydroxycamptothecin in human colon cancer cells [9]. Moreover, Deng et al. showed that the reduced expression of miR-506 is associated with tumor size, pathological tumor node metastasis (TNM) stage, and lymph node metastasis in 63 gastric carcinoma patient tumors [10], also consistent with our findings. However, they defined of Yes-associated protein 1 (Yap1) as a direct target gene for miR-506 [10]. Hence, it is obvious that miR-506 may have multiple targets that regulate tumorigenesis, growth, proliferation, migration, invasion and angiogenesis in gastric carcinoma.

Our microarray analysis revealed differential expression of miR-506 in gastric carcinoma tissues compared with NGT. RT-qPCR confirmed downregulation of miR-506 in gastric carcinoma tissues compared with NGT. These results indicated that miR-506 may serve as a tumor suppressor in gastric carcinoma. As miRNAs perform their biological functions by suppressing their target genes, identification of the target genes of miR-506 is important to clarify the functional mechanism of miR-506 in gastric carcinoma tumorigenesis. Our bioinformatics analysis revealed ZEB2 as a potential target gene of miR-506, and sequence analysis revealed that ZEB2 contains one putative miR-506 binding site within its 3′-UTR. Dual-luciferase reporter assays confirmed that miR-506 directly targeted the 3′-UTR region of the ZEB2 transcript in AGS cells.

ZEB2 is an important member of the ZEB family that induces endothelial-mesenchymal transition through repression of E-cadherin and promotes tumor development in gastric carcinoma [11–20]. High ZEB2 expression is associated with poor outcome in pancreatic cancer, oral squamous carcinoma, breast cancer, ovarian cancer, gastric cancer, colorectal carcinoma and lung cancer [20–33], which is related to its enhancement of proliferation, adhesion and signal transduction and other biological processes. Previous studies have also shown that ZEB2 is targeted by various miRNAs, including miR-200, miR-145, miR-132, MiR-338, miR-144, miR-139, miR-153, miR-218, miR-30, miR-124, miR-598, miR-454 and miR-374 [17, 30, 31, 34–47]. To the best of our knowledge, our study was the first to reveal the regulation of ZEB2 by miR-506 in gastric carcinoma. With limited number of the clinical cases, we were able to evaluate correlations between miR-506 and ZEB2 expression in gastric carcinoma specimens, suggesting that this regulation is strong. Furthermore, the association of miR-506 levels with patients’ prognosis demonstrates the clinical relevance of the current study.

We next evaluated the function of miR-506 in gastric carcinoma metastasis and invasion by a set of loss-of-function and gain-of-function experiments. We confirmed that upregulation of miR-506 significantly inhibited cell migration and invasion of AGS cells. In future studies, it may be interesting to study the miR-506 targeting of ZEB2 downstream signaling pathways, and the molecular mechanisms underlying the downregulation of miR-506 in gastric carcinoma. This newly identified miR-506/ZEB2 axis might be a promising diagnostic biomarker for gastric carcinoma patients and could be a potential therapeutic target in the management of gastric carcinoma.

## MATERIALS AND METHODS

### Protocols and samples

Twenty-six paired surgical specimens of gastric carcinoma tissues and their paired non-cancerous gastric tissues (more than 2.5 cm from the edge of the cancer tissue) were obtained from gastric carcinoma patients who underwent surgery between 2011 and 2013 at the First Affiliated Hospital of Jinzhou Medical University. All tissue samples were immediately frozen in liquid nitrogen after resection and stored at −80°C until RNA extraction. Informed consent was obtained from each patient or family. The study protocol was approved by the ethics committee of the First Affiliated Hospital of Jinzhou Medical University.

### Cell culture

A human gastric carcinoma cell line AGS was purchased from American Type Culture Collection (ATCC, Rockville, MD, USA), and was cultured in RPMI1640 medium (Invitrogen, Carlsbad, CA, USA) supplemented with 10% fetal bovine serum (FBS; Sigma-Aldrich, St Louis, MO, USA), 100 units/ml penicillin and 100 μg/ml streptomycin (Sigma-Aldrich, Shanghai, China). The human embryonic kidney 293 cell line was obtained from Saier Biotechnology Incorporated Company (Tianjing, China) and cultured in Dulbecco’s modified Eagle’s medium (DMEM, Invitrogen) supplemented with 10% FBS, 100 units/ml penicillin and 100 μg/ml streptomycin. Cells were cultured at 37°C in a humidified incubator with an atmosphere of 5% CO_2_.

### Microarray assay analysis

Total RNA, including miRNAs, was isolated with the miRNeasy Mini Kit (Qiagen, Germany) according to the manufacturer's protocol. RNA concentration, purity and RNA integrity number (RIN) were determined using a NanoDrop-2000 spectrophotometer (Peqlab, Erlangen, Germany), an Agilent 2100 Bioanalyzer and RNA 6000 Nano LabChip Kit (both Agilent Technologies, Santa Clara, CA, USA). The inclusion criteria for the sample to be acceptable for microarray analysis was a minimum RIN ≥7. MiRNAs were labeled by the miRNA Complete Labeling and Hyb Kit (Agilent Technologies, Chengdu, China), according to the manufacturer’s instructions. For Array hybridization, each slide was hybridized with 100 ng Cy3-labeled RNA in a hybridization oven at 55°C, 20 rpm for 20 hours, according to the manufacturer’s instructions. After hybridization, slides were washed in staining dishes (Thermo Shandon, Waltham, MA, USA) with a Gene Expression Wash Buffer Kit (Agilent Technologies). Afterwards, the slides were scanned by an Agilent Microarray Scanner (Agilent Technologies) and analyzed by Feature Extraction software 10.7 (Agilent Technologies) with default settings. Raw data were normalized by Quantile algorithm, Gene Spring Software 11.0 (Agilent Technologies). The differentially expressed miRNAs were determined by evaluating fold-change compared with non-cancerous matched tissue; miRNAs with a fold-change> 2 were categorized as upregulated miRNAs, and miRNAs with a fold-change <0.05 were categorized as downregulated miRNAs. The fold-change value was calculated to determine the extent and direction of differential expression between gastric carcinoma and the control. P values <0.05 were considered to be statistically significant. Fold change=log2 (gastric carcinoma/control).

### Cell transfections

MiR-506 mimics, antisense and control null constructs were obtained from RiboBio (Guangzhou, China). Cells were transfected with at a final concentration of 20 nM using Lipofectamine 3000 (Invitrogen), according to the manufacturer's instructions.

### Quantitative reverse transcription polymerase chain reaction (RT-qPCR)

Total RNA from cell lines and tissues was extracted with TRIzol^®^ reagent (Invitrogen), according to the manufacturer’s instructions. The concentration of the total RNA was quantified using a NanoDrop-2000 (Peqlab). cDNA was synthesized with the TaqMan MicroRNA reverse transcription kit (Applied Biosystems, Foster City, CA, USA) and expression was determined using the QuantMir RT Kit (System Biosciences, Mountain View, CA, USA) and ABI 7900 Sequence Detection System (Applied Biosystems) using miR-506 and U6 primers (Applied Biosystems). To evaluate ZEB2 mRNA expression, RNA was reverse-transcribed into cDNA by the Primescript^™^ RT reagent kit (Takara, Dalian, China) and examined with the Real-time PCR Mixture Reagent (Takara) and ABI 7900 Sequence Detection System (Applied Biosystems), using primers for ZEB2 and α-tubulin. The relative miR-506 expression was normalized to U6 expression and the expression of ZEB2 mRNA was normalized to α-tubulin using the 2-ΔΔCt method. Values of genes were first normalized against U6 or α-tubulin, and then compared to the experimental controls.

### Western blot analysis

At 24 hours after transfection, cells were washed with PBS and lysed in lysis buffer (Cell Signaling Technology, Danvers, MA, USA) for 30 minutes. A BCA kit (Pierce Biotechnology, Rockford, IL, USA) was used to determine protein concentrations. After gel electrophoresis, membrane transfer and blocked with 5% non-fat milk, the blots were probed with antibodies against ZEB2 (sc-271984; Santa Cruz Biotechnology, Inc., Santa Cruz, CA, USA) and α-tubulin (#2144; Cell Signaling, Beijing, China) overnight. The blots were then washed and incubated with horseradish peroxidase-conjugated secondary antibodies (Santa Cruz Biotechnology, Inc.) for 1 hour at room temperature, followed by development of the signal by an ECL detection reagent (Pierce, Shanghai, China) and quantification with densitometry using Image J software (Bethesda, MA, USA). Blotting images were representative from 5 repeats. α-tubulin was used as a protein loading control.

### Transwell migration and invasion assays

Cell migration and invasion were assessed using transwell assays. For migration, 5×10^4^ cells in 200 μl of 0.1% FBS-containing medium were placed in the upper chamber of an insert (pore size, 8 μm) (Becton-Dickinson Biosciences, San Jose, CA, USA) and the lower chamber was filled with 10% FBS (600 μl). For invasion, the same density of cells was placed into the upper chamber precoated with Matrigel (Becton-Dickinson Biosciences). After 48 hours’ incubation, cells on the upper chamber of the filter were removed with a cotton swab and then the cells on the underside were fixed with 4% paraformaldehyde, stained with 0.1% crystal violet in 20% ethanol, and counted in five randomly selected fields using a phase contrast microscope. Migrating cells were monitored by photographing at 200x magnification with a LEICA microscope (Darmstadt, Germany) in 5 independent fields for each well. The assays were performed in triplicate.

### Luciferase-reporter activity assay

Luciferase-reporters were successfully constructed using molecular cloning technology. Target sequence for ZEB2 miRNA 3'UTR clone was purchased from Creative Biogene (Shirley, NY, USA). Wild-type or the mutant ZEB2 3′-UTR were cloned into the PmirGLO vector. Cells (6×104) were seeded in 48-well plates and co-transfected with 400 ng of PmirGLO-ZEB2-WT or PmirGLO-ZEB2-MUT constructs along with miR-506 or as-miR-506 or null mimics using Lipofectamine 3000 reagent (Invitrogen). Luciferase activities were measured with the dual luciferase reporter kit (Biyuntian, Jiangsu, China) at 48 hours after transfection.

### Xenograft model in nude mice

The male nude mice at 10-week-old (SLAC Laboratory Animal Co. Ltd, Shanghai, China) were used for subcutaneous implantation of gastric carcinoma cells. AGS cells were resuspended in 50μl PBS and injected subcutaneously into the flanks of nude mice to establish the xenograft model (n=3 for each group). After 30 days, the mice were sacrificed and the size of the dissected tumor was measured. The tumor volume (V) was calculated by measuring the length (L) and width (W) and applying the formula V = (L × W 2) × 0.5.

### Statistical analysis

The data was statistically analyzed with GraphPad Prism 7 (GraphPad Software, Inc. La Jolla, CA, USA), as described [48–50]. The Student’s T test was performed to compare the data of 2 groups. Bivariate correlations were calculated by Spearman's Rank Correlation Coefficients. The values were expressed as mean ± standard deviation (SD). When *p*<0.05, the data was considered as significant. Kaplan-Meier curve was applied to record the overall survival of the patients included in this study.
